# Phylogenetic Structure Shifts Across Life-History Stages in Response to Microtopography and Competition in Subtropical Forests

**DOI:** 10.3390/plants14142098

**Published:** 2025-07-08

**Authors:** Weiqi Meng, Haonan Zhang, Lianhao Sun, Jianing Xu, Yajun Qiao, Haidong Li

**Affiliations:** 1 Innovative Research Team for Forest Restoration Mechanisms, Chishui National Ecological Quality Comprehensive Monitoring Stations, Nanjing Institute of Environmental Sciences, Ministry of Ecology and Environment (MEE), Nanjing 210042, China; 2 Co-Innovation Center for Sustainable Forestry in Southern China, Jiangsu Province Key Laboratory of Soil and Water Conservation and Ecological Restoration, Nanjing Forestry University, Nanjing 210037, China

**Keywords:** phylogenetic structure, plant community, life stage, microtopographic, subtropical forest

## Abstract

This study focuses on a subtropical evergreen broad-leaved forest in China, utilizing a large permanent plot established in the Yaoluoping National Nature Reserve. By integrating data from a full-stem census and total station surveying, we analyzed the phylogenetic structure of the plant community as a whole and across different life-history stages (saplings, juveniles, and adults) while quantitatively assessing microtopographic variables and an interspecific competition index. The results indicate that the overall community in the Yaoluoping plot exhibited a weakly overdispersed pattern, and key microtopographic factors—including aspect, terrain position index (TPI), terrain ruggedness index (TRI), roughness, and flow direction—significantly influenced the evolution of phylogenetic structure. Distinctions were also observed among saplings, juveniles, and adults in phylogenetic structuring across life-history stages. Specifically, saplings displayed a higher degree of phylogenetic clustering, significantly influenced by density, elevation, TPI, and flow direction—suggesting that environmental filtering predominates at this stage, possibly due to lower environmental tolerance, limited dispersal ability, and conspecific negative density dependence. In contrast, juveniles and adults showed a more dispersed phylogenetic structure, with density, interspecific competition, aspect, TRI, TPI, and roughness significantly correlated with phylogenetic patterns, indicating that competition and niche differentiation become increasingly important as trees mature and establish within the community. Interspecific competition was found to play a crucial role in community structuring: the competition index was generally negatively correlated with the net relatedness index (NRI) and nearest taxon index (NTI) in juveniles and adults, implying that intense competition leads to the exclusion of some species and reduces overall diversity, with the strength and significance of competitive effects differing across stages. This study enhances our understanding of the complex interplay between microtopography and interspecific competition in shaping the phylogenetic structure and diversity of subtropical evergreen broad-leaved forests, elucidates the coupled mechanisms among microtopography, phylogenetic structure, and competition, and provides a scientific basis for forest conservation and management.

## 1. Introduction

Under mounting pressures from global climate change, sharp declines in biodiversity, and increasing human societal demands, forest ecosystems have become an important frontier in terrestrial ecological research [1,2,3]. Forests not only play a fundamental role as regulators in the global carbon cycle but are also crucial for maintaining biodiversity and delivering multiple ecosystem services [1]. In recent years, dramatic increases in atmospheric carbon dioxide concentrations and shifting climatic patterns have brought new attention to the carbon sink function of forests and the stability of community structure and function, especially in the face of anthropogenic disturbance [4,5]. Concurrently, habitat fragmentation and biodiversity loss driven by deforestation and land-use change have accelerated worldwide, elevating the urgency for sustainable forest management [6,7]. Against this backdrop, advancing our understanding of forest diversity and its assembly mechanisms not only uncovers the scientific rules behind community succession and ecosystem stability but also lays a solid foundation for effective conservation and management [8,9].

The structure and function of forest communities result from the interplay of multiple ecological processes. Large-scale environmental gradients, such as latitude, elevation, and climatic zones, have long been recognized as key drivers of community composition and species distribution patterns [9,10,11]. However, with advances in data collection and analytical techniques, increasing attention has shifted to finer-scale ecological processes. Some studies reveal the local heterogeneity of forest carbon sinks, showing how elevation, slope, and aspect strongly influence carbon storage and community structure [1,12]. Others incorporate environmental filtering, competitive exclusion, and neutral processes into comprehensive frameworks to unravel plant community dynamics under different environmental conditions [13,14,15,16]. These works suggest that community assembly cannot be wholly explained by niche or neutral theory alone but is driven by an integrated set of factors, including natural selection, species dispersal, and stochastic processes [17,18,19].

In the study of forest diversity and its drivers, phylogenetics has rapidly emerged as an important and integrative discipline, giving rise to phylogenetic ecology [12,14,20,21]. Relative to traditional approaches emphasizing species richness or functional traits, phylogenetic metrics—such as phylogenetic diversity (PD)—measure the evolutionary distinctiveness among community members and offer insights into community structure and succession from a macroevolutionary perspective [22,23]. Indices such as PD, the net relatedness index (NRI), and the nearest taxon index (NTI) further quantify the relative contributions of environmental filtering, interspecific competition, or neutral processes to community assembly [15,24,25,26]. For example, phylogenetic clustering within a community typically implies the dominance of environmental filtering for similar ecological niches or conserved traits, while phylogenetic overdispersion suggests that density dependence or interspecific competition facilitates coexistence [27,28]. Nevertheless, most such studies focus on idealized or relatively homogenous settings, and few have thoroughly addressed the complexity introduced by microtopographic heterogeneity [15,29].

Microtopography, referring to fine-scale landform variations (typically from several to tens of meters), such as small ridges or depressions, slope variations, subtle hydrological flow, or soil micro-structure differences [15,16,30], can regulate the distribution of light, moisture, and nutrients at the surface with surprising effectiveness [15,16]. For instance, south-facing slopes often receive more solar radiation and have lower soil moisture, while north-facing slopes experience higher humidity and reduced light [15,16,31]. Runoff accumulation in depressions can alter soil oxygenation and nutrient cycling [7]. Such microtopographic variability creates a mosaic of microhabitats, potentially modifying competition and resource acquisition strategies, and thereby influences community phylogenetic structure [10]. By integrating phylogenetic structure with terrain complexity, it is possible to discern the spatial scales at which environmental filtering and interspecific competition hold sway [20,32]. Some studies find competition is more important at very small scales (e.g., 2.5 m × 2.5 m), while strong environmental filtering emerges at larger scales [19,27], but such conclusions require empirical validation across diverse landscapes and floras [9]. Meanwhile, phylogenetic structure may also shift through time as succession proceeds: early filtering might privilege trait conservatism, while later-stage competition among similar species drives communities toward randomness or overdispersion [6,33]. Superimposed on microtopographic heterogeneity, community assembly thus displays increased spatial and temporal complexity [12].

Phylogenetic ecology has become a key approach to deciphering community diversity patterns. Since Webb et al. first aligned phylogenetic diversity with community ecological studies [14,23], related theories and methodologies have flourished. PD [22] quantitatively reflects evolutionary history among coexisting species; the NRI and NTI characterize whether species are clustered or overdispersed in the phylogeny [25,26]. Prior studies in tropical rainforests, temperate deciduous forests, and other regions have demonstrated the efficacy of phylogenetic indices for uncovering community assembly mechanisms [9,24]. Increasing theoretical and empirical evidence shows that phylogenetic relationships can shape not only trait similarity but also population dynamics, as closely related species often share similar resource requirements, environmental adaptations, and responses to environmental filtering or competition [34,35,36,37]. Coevolutionary interaction and environmental filtering can reinforce demographic similarities among phylogenetically close species [14,23,25,38]. Thus, phylogenetic structure not only reflects the distribution of functional traits but also underpins patterns of population growth, survival, and interspecific competition in community assembly [25,39,40,41]. Notably, in biodiversity hotspots such as Southeast Asia and the South American Andes, clustering often reflects strong environmental filtering [4], but in some areas, close relatives are excluded through competition, resulting in overdispersion [28]. Neutral stochastic processes may also be influential during species formation and community reassembly [13]. Divergences in results and interpretations arise from differences in geological history, species pool size, successional stage, and scaling effects [9,24]. In heterogeneous regions, topographic complexity further challenges any single-theory explanation [15,16,42]. Initially, microtopography research focused on erosion monitoring and hydrological modeling, but more recent work addresses microtopographic effects on plant diversity maintenance and functional responses [30,43]. Studies in temperate forests of North America and Europe demonstrate that microtopography drives fine-scale soil moisture, pH, and nutrient gradients, influencing the spatial patterns of both saplings and adults [44,45]. Likewise, in subtropical and tropical forests, high rainfall and topographic complexity foster a dynamic interplay among environmental filtering and competitive exclusion [15,16,30,31]. Yet, comprehensive studies integrating microtopography and forest phylogenetic structure remain rare. Prior work mostly addresses topographic position or aspect effects on stand density or composition, with limited attention to scaled patterns in PD, the NRI, or the NTI or to the links between microtopography and competition intensity (Hegyi index). Whether competitive effects are consistent across scales and how competition and filtering interact across life stages remain insufficiently explored [46].

Against this background, the current study focuses on subtropical evergreen broad-leaved forests in the Yaoluoping National Nature Reserve, Anhui, conducting full-stem censuses and high-resolution spatial mapping at multiple scales across 11.56 ha. Using the V.PhyloMaker 2 package [47], we constructed a comprehensive phylogeny and calculated indices (PD, NRI, NTI, and competition index [CI]) to address the following questions: (1) Do the NRI and NTI vary significantly across spatial grains? At which scale and under what conditions do microtopographic variables and competition drive changes in phylogenetic structure? (2) Does the balance between environmental filtering and interspecific competition shift across life-history stages (sapling, juvenile, and adult), and are there transitions from ‘clustering to overdispersion’ or from ‘overdispersion to randomness’? (3) How can an integrated microtopography and phylogenetic perspective clarify the interplay between filtering and competition during succession and inform sustainable forest conservation and management [5,18]? Addressing these questions will deepen our understanding of meso- and small-scale assembly dynamics and expand theoretical and practical frameworks for biodiversity conservation and adaptive management in heterogeneous environments [24,30].

## 2. Materials and Methods

### 2.1. Study Area and Censuses

**Figure 1:**
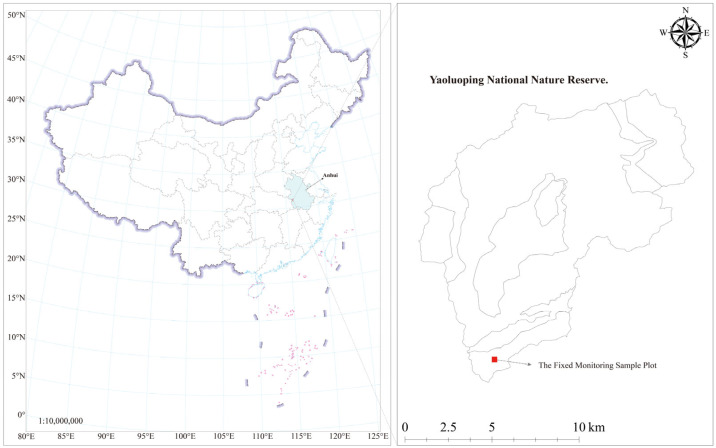
Regional map of the fixed monitoring sample plot in the Yaoluoping National Nature Reserve, and the light blue area in the figure indicates the province where the sample site is located, Anhui Province. The base map of China (Approval Number: GS(2023)2765) was obtained from the Ministry of Natural Resources Standard Map Service System (https://www.mnr.gov.cn/, accessed on 12 May 2025), and the text content has been modified to better suit the research context.

The study site is in the Yaoluoping National Nature Reserve, Yuexi County, Anhui Province, China, within the core protected area (30°57′46.11″–30°57′47.81″ N, 116°04′41.33″–116°04′41.08″ E). The 340 m × 340 m (11.56 ha) plot lies at the southeastern margin of the Dabie Mountains, with minimal human disturbance (Figure 1). The region features diverse landforms—including low and medium mountains, valleys, and basins—shaped by orogenic and exogenic processes. The climate is subtropical monsoon, transitional between the warm-temperate and northern subtropical zones, with abundant rainfall and pronounced humidity advected from the lower Yangtze. Soils are heterogeneous, comprising yellow-brown earth, brown earth, mountain meadow soil, and marsh soil. The vegetation is a mosaic of evergreen and deciduous broad-leaved forest, with warm-temperate elements increasing with elevation.

Following CTFS protocols, the plot was divided into 289 quadrats (20 m × 20 m) [48]. Each was subdivided into 5 m × 5 m working quadrats for census, totaling 4624 subplots (Figure S1 in the Supplementary Files). All trees with a diameter at breast height, measured at 1.3 m above ground (DBH), greater than 1 cm were identified to the species level and measured for DBH, height, and spatial position. The spatial position of each tree was geolocated with Real-Time Kinematic GPS (RTK GPS). Additionally, when trees had branches at the measurement height, the main stem and branches at 1.3 m needed to be identified and differentiated, the number and positions of branches were recorded, and the DBH of each branch was measured separately to accurately record the growth status of individual stems (Figure S2 in the Supplementary Files).

All stems were assigned to their respective working quadrat by spatial position and code. For finer-scale analyses, each 5 m × 5 m plot was further divided into four 2.5 m × 2.5 m quadrats using the same orientation and coding scheme; a 10 m × 10 m scale was created by merging four neighboring 5 m quadrats.

### 2.2. Microtopographic Variables

**Figure 2:**
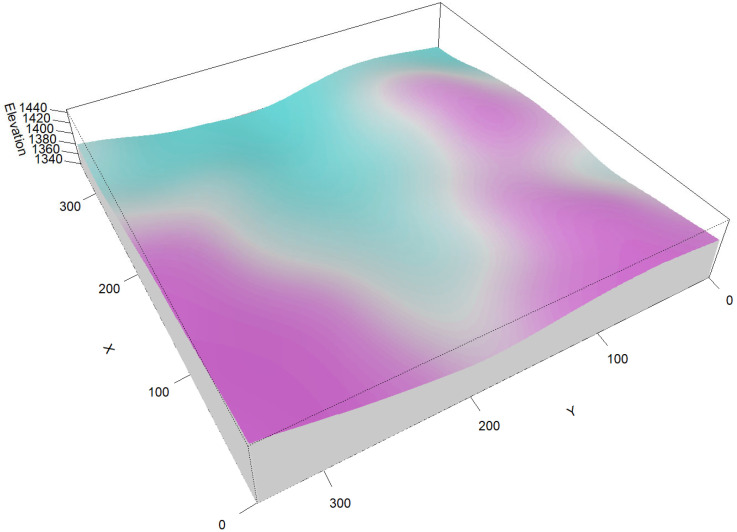
The microtopography of the fixed sample plot in Yaoluoping shows the elevation of the sample plot and the comprehensive topographic conditions. These maps were generated using the Epanechnikov kernel function of the terra package in R (version 4.4.2), and the intensity values range from light blue (low) to light purple (high).

For each of the 4624 working quadrats, total station data were used to measure slope, aspect, elevation, and vertex positions. These data produced a digital elevation model (DEM) of the plot (Figure 2), enabling the quantification of elevation, slope, aspect, the TPI, the TRI, roughness and flow direction for each tree’s plot position (Figure S3 in the Supplementary Files).

In our research, numerical values for aspects assigned higher scores to south-exposed/high-irradiance slopes and lower scores for north-exposed/shaded aspects. The TPI measures the difference in elevation of a focal cell relative to its neighbors, while the TRI quantifies the standard deviation of the elevation among adjacent cells. Flow direction (Flowdir) represents the hydrological potential for water accumulation, and it is determined by the elevation differences between a given pixel and its adjacent pixels, representing the primary flow path of water accumulation. A higher flow direction value signifies a greater capacity for water convergence, indicating elevated soil moisture levels in that area.

Considering that there are significant differences in the order of magnitude among different microtopographic factors, all indices were standardized (z scores). Calculations were performed in R (version 4.4.2) using the spatstat and terra packages [49].

### 2.3. Phylogenetic Tree Construction

A phylogenetic tree for the 104 species (39 families, 61 genera) was constructed using the V.PhyloMaker 2 package [47] in R (version 4.4.2). This package is based on the phylogenetic tree of world vascular plants, GBOTB, published by Smith and Brown [50], and the phylogenetic tree of ferns published by Zanne [51]. The integrated skeleton was revised and supplemented to form GBOTB.extended.tre, which contains a total of 74,533 species and has time calibration. To construct the phylogenetic tree of the plant community in the sample plot, the species within the sample plot were matched with the species in GBOTB.extended.tre, and the phylogenetic tree was pruned according to the checklist.

Firstly, it was necessary to standardize the names of tree species using the plantlist package in R (version 4.4.2) before constructing the phylogenetic tree [52]. Then, the phylogenetic tree of the plant community was generated via the phylo.maker function of the V.Phylomaker 2 package, following the scenario parameter “S3”. Finally, the ggtree package was used to visualize and annotate the phylogenetic tree [53].

### 2.4. Phylogenetic Index and Neighborhood Effects Variables

Phylogenetic diversity (PD), the NRI, and the NTI quantified the structure and evolutionary relatedness of the community. PD follows Faith’s index [22], reflecting the total phylogenetic branch lengths of the species in a community (Figure S3b–d in the Supplementary Files). The NRI and NTI measure standardized effect sizes for the mean pairwise phylogenetic distance (MPD) and mean nearest-taxon distance (MNTD), respectively [14,20]. SR denotes species richness at each spatial scale.

The competition index (*CI*) followed Hegyi’s model [54], where each tree was the focal individual, and the *CI* was computed as follows:

(1) $$CI=\sum_{j}^{n}\frac{d_{j}}{d_{i}{dist}_{ij}}$$

where *d_i_* is the focal tree DBH, *d_j_* is the DBH of neighboring competitors within the specified scale, and *dist_ij_* is their spatial distance. Calculations used the R packages spatstat and picante [39,49].

The SR index represents species richness, which reflects the situation of species diversity within small sample plots at different local scales (2.5 m, 5 m, and 10 m), and it is mainly statistically analyzed according to the specified specifications of small sample plots at three different scales (Figure S3a in the Supplementary Files).

### 2.5. Effects of Microtopography and Neighborhood Competition Factors

To assess the influences of microtopographic variables and the CI at three different scales, linear mixed-effects models were fit with SR, PD, the NRI, or the NTI as response variables and the CI, elevation, aspect, slope, TPI, TRI, and flow direction as predictors. For the NRI and NTI models, the SR factor was supplemented in addition to the existing seven independent variables.

Tree life stage was categorized as sapling (dbh < 5 cm), juvenile (5 ≤ dbh < 15 cm), or adult (dbh ≥ 15 cm) [55]. Models for each life stage quantified how microtopography and competition influenced local diversity and phylogenetic structure. Models were constructed as follows:

(2) $$\begin{array}{cc} {\left(SR/PD\right)}_{scale}= & \beta_{0}+\beta_{1}\times {CI}_{scale}{+\beta }_{2}\times {Elevation}_{scale}+\beta_{3}\times {Aspect}_{scale}+\beta_{4}\times {Slope}_{scale}\\ & +\beta_{5}\times {TPI}_{scale}+\beta_{6}\times {TRI}_{scale}+\beta_{7}\times {Flowdir}_{scale}+\varepsilon \end{array}$$

(3) $${\left(NRI/NTI\right)}_{scale}=\beta_{0}+\beta_{1}\times {CI}_{scale}{+\beta }_{2}\times {Elevation}_{scale}+\beta_{3}\times {Aspect}_{scale}+\beta_{4}\times {Slope}_{scale}\;+\beta_{5}\times {TPI}_{scale}+\beta_{6}\times {TRI}_{scale}+\beta_{7}\times {Flowdir}_{scale}+\beta_{8}\times {SR}_{scale}+\varepsilon$$

In the aforementioned two formulae, the microtopographic variables, phylogenetic diversity indices, and interspecific competition indices all represent the influence under the same sampling scales, which include 2.5 m, 5 m, and 10 m. The coefficients from *β*_1_ to *β*_7_ serve to determine the extent of the direct influence that each microtopographic predictor has on these responses. The random-effects framework includes ε, which captures the species-specific baseline disparities as well as the variable nature of the responses. Species constitute the random-effect part of the model. The model is capable of automatically adjusting the random intercepts and slopes of different species. This effectively enhances the stability and universality of the model and also reduces the systematic biases caused by the differences among species or the differences in life-history stages. By establishing the model, it is possible to effectively combine multiple different topographic variables (abiotic factors) or interspecific relationship variables (biotic factors) to explain the complex ecological processes influenced by multiple factors. All analyses were conducted in R (version 4.4.2) using the lme4 package [56].

## 3. Results

### 3.1. Community-Wide Phylogenetic Structure

**Figure 3:**
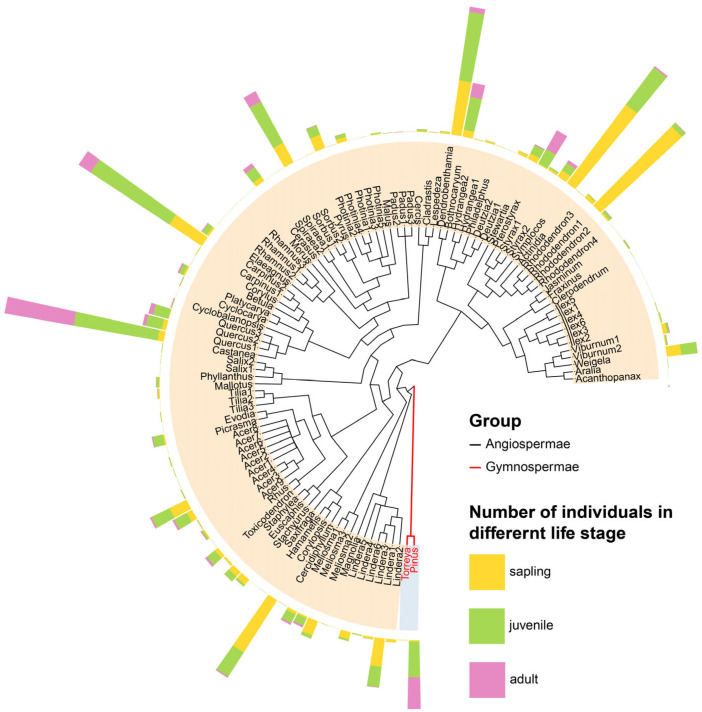
Phylogenetic relationships of species in the Yaoluoping plot community. Species are coded by abbreviation (genus + number); full names are available in Supplementary Table S1. The height of each bar in the outer ring represents the number of individuals per species in each life-history group.

**Figure 4:**
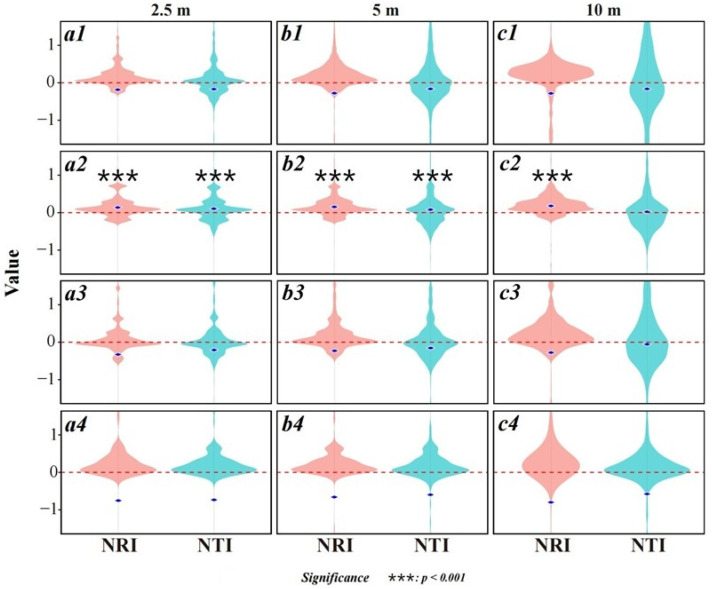
Community net relatedness index (NRI, the red-marked violin plot) and nearest taxon index (NTI, the blue-marked violin plot) at three different scales across life-history stages in the Yaoluoping plot. (**a1**–**c1**) The whole plant community; (**a2**–**c2**) sapling stages; (**a3**–**c3**) juvenile stages; (**a4**–**c4**) adult stages. Blue dots indicate mean NRI/NTI values; the red dashed line (value = 0) is the null model. Values > 0 indicate clustering, and values < 0 indicate overdispersion. Detailed significance is provided in Supplementary Table S2.

The census recorded 104 tree species (39 families, 61 genera), predominantly deciduous broad-leaved trees, with *Castanea seguinii*, *Carpinus turczaninowii*, *Cornus kousa*, and *Symplocos paniculata* as the dominant species. Among them, all but two (*Pinus huangshanensis* and *Torreya grandis*) were angiosperms, predominantly of Rosaceae, Sapindaceae, Lauraceae, and Aquifoliaceae. The community was dominated by saplings and juveniles, with relatively few adults (Figure 3). Overall, the community NRI and NTI values were less than zero at all three spatial grains (2.5 m, 5 m, and 10 m), indicating a trend toward phylogenetic overdispersion, but the differences were not statistically significant (Figure 4(a1–c1)). In all scale treatments, the NRI and NTI were near the null model expectations, demonstrating a largely random phylogenetic structure.

Life-history stage analysis showed that, in the sapling stage, both the NRI and NTI were significantly greater than zero at all scales, evidencing strong phylogenetic clustering (Figure 4(a2–c2)). For juveniles and adults, the NRI and NTI values fell below zero at all scales, suggesting overdispersion, but this trend did not reach statistical significance (Figure 4(a3–c3,a4–c4)).

### 3.2. Effects of Microenvironmental Factors and Competition on Phylogenetic Structure

**Figure 5:**
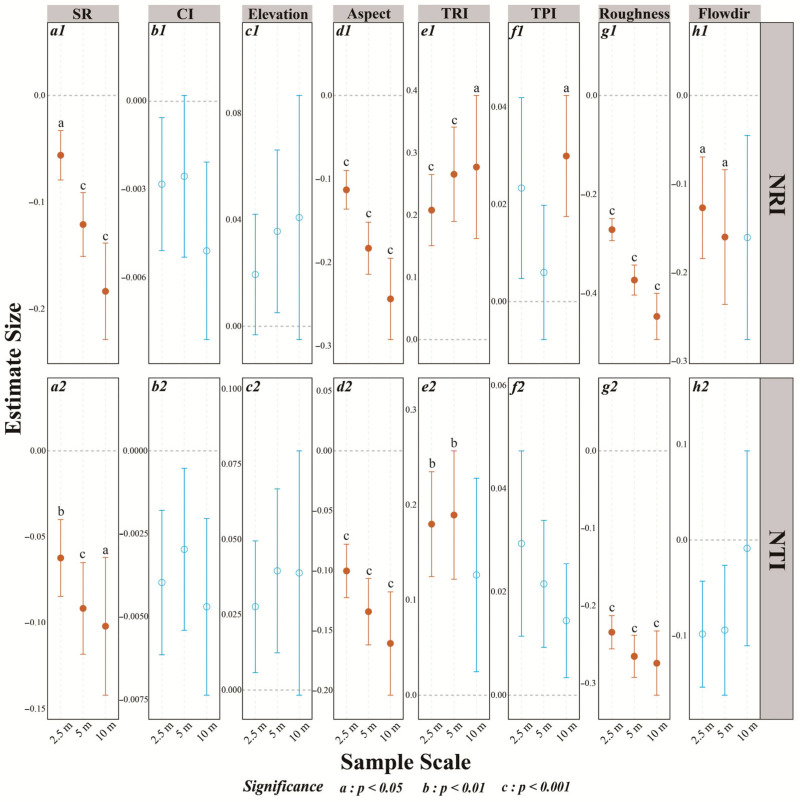
Parameter estimates for the effects of microtopographic factors, the interspecific competition index (CI), and species richness (SR) on the net relatedness index (NRI, **a1**–**h1**), and nearest taxon index (NTI, **a2**–**h2**) across three different scales. The circles represent estimated effects, with error bars indicating standard deviations. The red error bars and solid filled circles denote statistically significant results, whereas the blue error bars and hollow circles indicate non-significant findings. The dashed line at zero indicates no effect. Microtopographic factors include elevation, aspect, the terrain position index (TPI), the terrain ruggedness index (TRI), roughness, and flow direction (Flowdir).

Modeling results demonstrated that species richness (SR), aspect, flow direction, roughness, and the TRI significantly impacted the NRI and NTI at different spatial grains (Figure 5(a1–h1,a2–h2)). Notably, SR, aspect, and roughness had negative effects (Figure 5(a1,a2,d1,d2,g1,g2)), and the magnitude of these effects increased with spatial scale. In contrast, the TRI exerted a positive influence on both the NRI and NTI, with the effect size on the NRI increasing with grain and a significant effect on the NTI only at 2.5 m and 5 m, not at 10 m. Flow direction was significantly negatively correlated with the NRI at 2.5 m and 5 m only (Figure 5(h1)), and correlations with the NTI or at 10 m were not significant (Figure 5(h1)). The TPI was only positively correlated with the NRI at the 10 m grain (Figure 5(f1)). The competition index (CI) showed general negative relationships with both the NRI and the NTI, though these did not reach statistical significance (Figure 5(b1,b2)).

**Figure 6:**
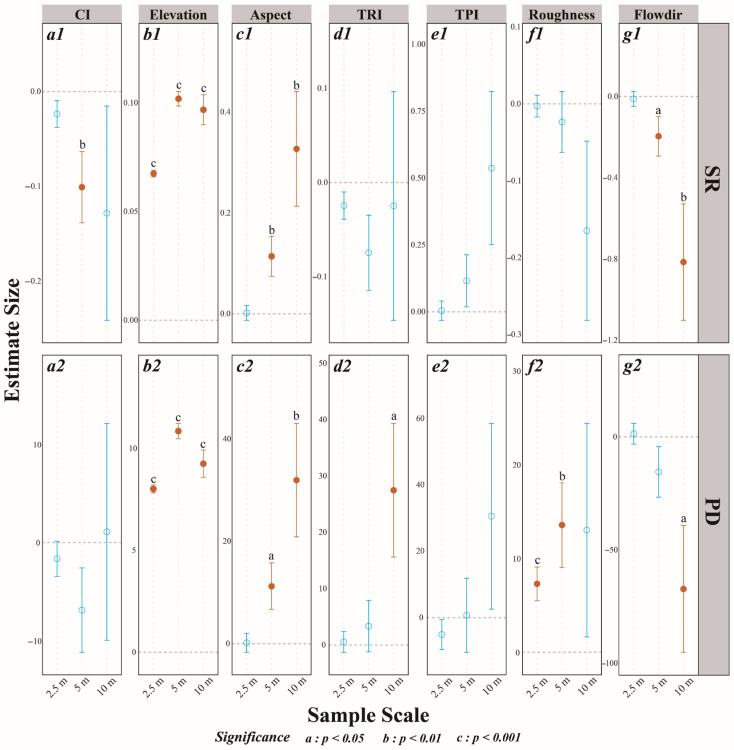
Parameter estimates for the effects of microtopographic factors and the interspecific competition index (CI) on species richness (SR, **a1**–**g1**) and phylogenetic diversity (PD, **a2**–**g2**) across three different scales. Significance: a: *p* < 0.05; b: *p* < 0.01; c: *p* < 0.001. The circles represent estimated effects, with error bars indicating standard deviations. The red error bars and solid filled circles denote statistically significant results, whereas the blue error bars and hollow circles indicate non-significant findings. The dashed line at zero indicates no effect. Microtopographic factors include elevation, aspect, the terrain position index (TPI), the terrain ruggedness index (TRI), roughness, and flow direction (Flowdir).

Regarding SR and PD, key effects came from elevation, aspect, roughness, and flow direction (Figure 6(a1–g1,a2–g2)). Both PD and SR increased significantly with elevation, with effect size growing then declining as grain increased (Figure 6(b1,b2)). Aspect had significantly positive effects on SR and PD only at the 5 m and 10 m scales (Figure 6(c1,c2)). Roughness was positively correlated with PD only at 2.5 m and 5 m (Figure 6(f2)), whereas flow direction negatively affected SR only at 5 m and 10 m (Figure 6(g1)). The CI affected SR significantly only at 5 m and did not have significant effects on PD at any grain (Figure 6(a1,a2)).

### 3.3. Effects of Microtopographic Factors and Interspecific Competition Across Life-History Stages

**Figure 7:**
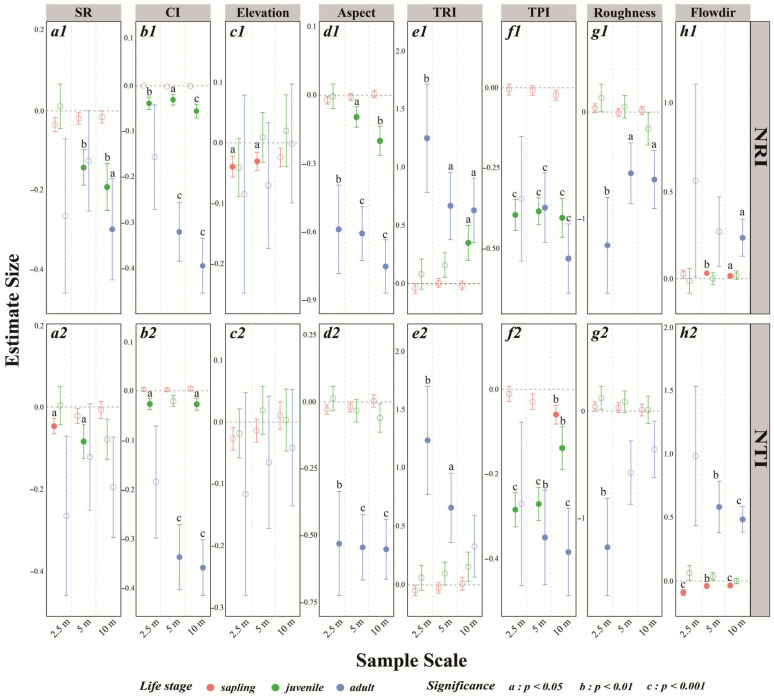
Parameter estimates for the effects of microtopographic factors, the interspecific competition index (CI), and species richness (SR) on the net relatedness index (NRI, **a1**–**h1**) and nearest taxon index (NTI, **a2**–**h2**) across different life-history stages and spatial scales. Significance levels: a: *p* < 0.05; b: *p* < 0.01; c: *p* < 0.001. The circles represent estimated effects, with error bars indicating standard deviations. Solid filled circles denote statistically significant results, whereas hollow circles indicate non-significant findings. The dashed line at zero indicates no effect. Microtopographic factors include elevation, aspect, the terrain position index (TPI), the terrain ruggedness index (TRI), roughness, and flow direction (Flowdir).

The sensitivity of phylogenetic structures in saplings, juveniles, and adults to specific microtopographic factors and competition in the community gradually increases from saplings to juveniles and then to adults. Particularly at the adult stage, the strongest responses to competition and terrain are observed, as indicated by the NRI and NTI (Figure 7(a1–h1,a2–h2)).

Overall, the NRI and NTI values for juveniles and adults show significant correlations with several key factors, including the competition index (CI), aspect, TRI, TPI, and roughness (Figure 7(b1,d1,e1,f1,g1,b2,d2,e2,f2,g2)). Specifically, the CI, aspect, and TPI tend to have negative effects, indicating that increased competitive intensity, more south-facing slopes, and higher terrain positions are generally associated with reduced phylogenetic clustering or increased overdispersion. In contrast, the TRI (terrain ruggedness index) generally exerts a positive effect, suggesting that greater local terrain heterogeneity can increase phylogenetic clustering.

Interestingly, hydrological factors represented by flow direction (Flowdir) significantly impact phylogenetic structure only during the sapling and adult stages, and the direction of the effect is inconsistent in saplings: for saplings, Flowdir has a positive effect on the NRI but a negative effect on the NTI (Figure 7(h1,h2)). This pattern implies that water accumulation may facilitate phylogenetic clustering with respect to deeper evolutionary relationships (NRI), while promoting overdispersion among the most closely related taxa (NTI). The competition index (CI) primarily affects juveniles and adults, showing a consistent negative correlation (Figure 7(b1,b2)), supporting the hypothesis that increased competition intensifies phylogenetic overdispersion in later life stages.

Species richness (SR) is strongly negatively correlated with the NTI in the sapling and juvenile stages, as well as with the NRI in the juvenile and adult stages. However, these negative correlations vary across spatial scales: the negative correlation between SR and the NTI in saplings is significant at the 2.5 m scale (Figure 7(a2)); for juveniles, the effect is significant only at the 5 m and 10 m scales; and for adults, SR is significantly related to the NRI only at the 10 m scale (Figure 7(a1)).

**Figure 8:**
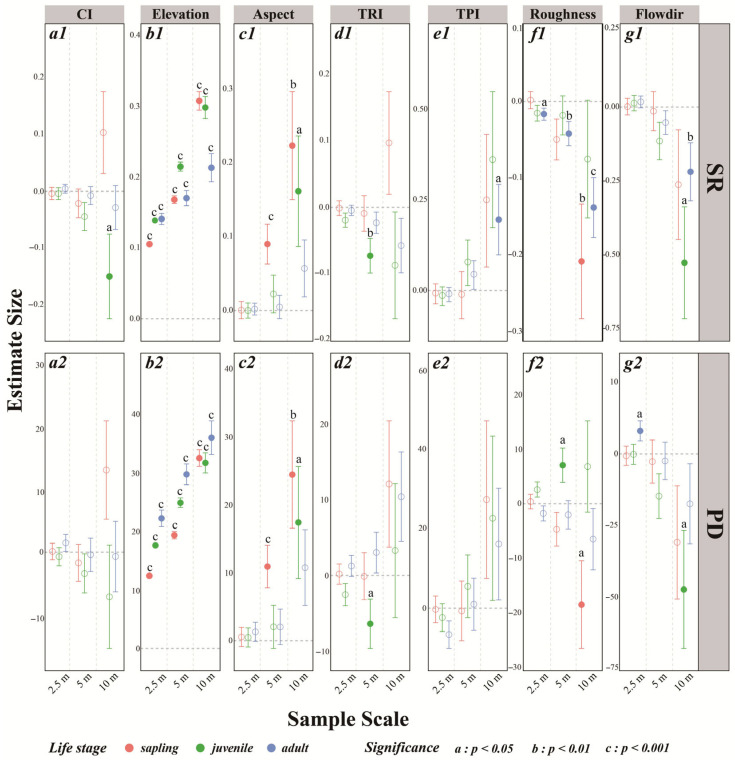
Parameter estimates for the effects of microtopographic factors and the interspecific competition index (CI) on species richness (SR, **a1**–**g1**) and phylogenetic diversity (PD, **a2**–**g2**) across different life-history stages and spatial scales. Significance levels: a: *p* < 0.05; b: *p* < 0.01; c: *p* < 0.001. The circles represent estimated effects, with error bars indicating standard deviations. Solid filled circles denote statistically significant results, whereas hollow circles indicate non-significant findings. The dashed line at zero indicates no effect. Microtopographic factors include elevation, aspect, the terrain position index (TPI), the terrain ruggedness index (TRI), roughness, and flow direction (Flowdir).

For both phylogenetic diversity (PD) and species richness (SR), elevation exerts a consistently significant positive effect across all life-history stages (Figure 8(a1–g1,a2–g2)). Aspect is particularly influential in the early stages of community succession, but its impact is only significant at larger spatial grains (5 m and 10 m). In these cases, higher aspect values, corresponding to more southerly orientations and greater sunlight exposure, are associated with increased SR and PD (Figure 8(c1,c2)). This result supports the well-established positive relationship between light availability and both phylogenetic and species diversity.

Roughness has an increasingly negative effect on SR and PD among saplings as spatial scale increases, becoming statistically significant only at the 10 m scale. For adults, roughness also negatively impacts SR, and this relationship is linearly correlated with spatial scale (Figure 8(f1,f2)). Flowdir demonstrates a strong negative effect on both juveniles and adults, with the impact being greatest on juveniles (Figure 8(g1,g2)). The CI only exerts a significant negative effect on SR for juveniles at the 10 m spatial scale, and effects at other scales and stages are not statistically significant (Figure 8(a1)).

## 4. Discussion

In this study, we explored the community structure and underlying dynamic mechanisms of a subtropical evergreen broad-leaved forest in the Yaoluoping National Nature Reserve, Anhui Province, from the dual perspectives of phylogenetic ecology and microtopography. By quantitatively analyzing the effects of microtopographic factors (elevation, aspect, slope, TPI, TRI, and flow direction) and interspecific competition intensity (CI) on community diversity indices (SR, PD, NRI, and NTI), we elucidated the patterns of phylogenetic structure evolution at fine spatial scales (2.5–10 m).

### 4.1. Stochasticity of Community Phylogenetic Structure and Its Drivers

Our findings indicate that the phylogenetic structure of the Yaoluoping forest community is overall characterized by stochasticity, suggesting the concurrent operation of environmental filtering and competitive exclusion. Specifically, while the NRI and NTI tended to be negative across all spatial scales, their values fluctuated around zero rather than consistently diverging significantly. This randomness suggests a dynamic interplay between environmental filtering and competition during community assembly [20]. In early successional stages, environmental filtering typically prevails, retaining species adapted to similar habitats and fostering phylogenetic clustering [27]. Over time, competition intensifies among functionally similar species, potentially driving a shift toward phylogenetic overdispersion or randomness [28]. NRI and NTI values near the null model indicate that, at this successional stage, the community does not exhibit pronounced clustering or overdispersion, reflecting the heterogeneous and temporally shifting influence of multiple processes across habitats and stages. The high heterogeneity created by shaded north slopes, sun-exposed south slopes, and low-lying terrains across the plot likely further explains the absence of a single dominant pattern, resulting in weak clustering or weak overdispersion [29,42].

Different microtopographic factors and competition exert varied influences on phylogenetic structure. We found that aspect had a significant negative effect on both the NRI and NTI at fine to intermediate scales, particularly for juveniles and adult trees, indicating that south-facing slopes tend to weaken phylogenetic clustering and drive the community toward overdispersion. This likely results from higher irradiance, greater evapotranspiration, elevated temperatures, and lower soil moisture on south slopes, intensifying competition [15]. In contrast, mesic shaded north slopes have higher soil moisture and reduced light, which lessens competitive intensity and favors the clustering of shade-tolerant taxa [57]. At a larger scale, the effect of slope aspect may be overridden by stronger gradients such as elevation or the TPI [29,42].

The TRI and TPI reflect terrain roughness and local relief, respectively [58]. At a smaller scale, higher TRI and TPI values substantially increase environmental heterogeneity (Figure 5(e1,f1,e2,f2)), thereby influencing phylogenetic structure by creating patchy microhabitats with variable soil moisture and nutrient availability [24]. High TRI or TPI value can enhance clustering and local diversity, with outcomes closely tied to species traits and root-associated microbes [59]. Our finding that the effect of the TRI diminishes at the 10 m scale (Figure 5(e1,e2)) supports the hypothesis that microtopographic control is strongest at meter-to-decimeter grains and becomes blurred at broader scales where macro-environmental gradients dominate [46,60].

Flow direction characterizes the direction of water aggregation and, thus, potential hydrological gradients. The frequent negative relationships of Flowdir with the NRI and NTI (Figure 5(h1,h2)) suggest that in wetter, low-lying areas, filtering and/or competition among closely related species is intensified, leading to greater overdispersion or randomness. Abundant water is likely to allow more species to coexist but also intensifies competition for resources such as light or nutrients [29,42]. When close relatives with similar niches prevail, competitive exclusion is enhanced; conversely, in drier habitats, differences in water-use strategies tend to foster clustering [31,32]. This highlights the strong coupling among moisture, light, temperature, and nutrients in shaping different ecological responses across space and time [13].

Finally, our scale-dependent findings emphasize the necessity of multi-scale tests. At small (2.5 m, 5 m) scales, increasing roughness or TRI tends to enhance the NRI and NTI (Figure 5(e1,g1,e2,g2)), underscoring the role of local heterogeneity in structuring species distributions and competitive dynamics [32]. Rugged terrain can create a variety of microhabitats—illuminated ridges and moist depressions [15,16]—favoring the clustering of niche-conservative species under filtering processes [17]. If resources are distributed more evenly at the micro-scale, competition is probably dampened, allowing for greater coexistence [24]. However, these relationships weaken at the 10 m scale, as local topographic differences are diluted by larger-scale heterogeneity [27]. This finding aligns with studies in temperate North American forests that demonstrate topography regulates sapling recruitment and species aggregation at a small scale [61,62], whereas broader gradients govern landscape diversity patterns.

### 4.2. Ontogenetic Shifts in the Coupled Effects of Environmental Filtering and Competition

Community phylogenetic structure emerges from the interplay between deterministic, niche-based processes and stochastic events [14]. A central mechanism driving deterministic patterns is phylogenetic niche conservatism: closely related species tend to share similar ecological traits. When environmental filtering is strong, only species bearing those conserved traits can persist under particular abiotic conditions, yielding phylogenetic clustering [63].

In early successional stages, abiotic resource requirements—light, temperature, soil moisture, and nutrients—play a dominant role in community assembly [64]. In our study, higher aspect values indicate more southerly exposure and greater insolation. Aspect was significantly positively correlated with SR and PD in saplings and juveniles (Figure 8(c1,c2)), reinforcing the notion that insolation promotes early-stage species richness and phylogenetic diversity and that south-facing slopes harbor higher diversity—a widely observed pattern [65,66]. As communities develop, phylogenetic structure shifts markedly across life stages. Standardized effect sizes of the net relatedness index (NRI) and nearest taxon index (NTI) indicate strong clustering in the sapling stage, which declines rapidly in juveniles and adults (Figure 4(a2–c2,a3–c3,a4–c4)). Such clustering reflects the combined influence of initial resource filtering and density-dependent effects, favoring closely related species with conserved reproductive and establishment traits—seed size, germination requirements, and dispersal modes—that determine which regional pool species can colonize successfully [17,19].

During later growth, interspecific competition intensifies. Limiting similarity processes drive phylogenetic overdispersion by excluding ecologically similar, closely related species under high competitive pressure [35,63]. Moreover, phylogenetically conserved resource-use strategies mediate the strength of negative interactions among relatives [67], facilitating the transition to overdispersed assemblages (NRI < 0, NTI < 0). This pattern aligns with negative density dependence, whereby abundant kin suffer greater biotic stress—pests, pathogens, or resource depletion—while specialized enemies further reinforce repulsion among close relatives [68]. Simultaneously, stochastic dispersal and drift processes establish a null expectation against which deterministic signatures can be tested [13]. Ultimately, the observed phylogenetic structure depends on spatiotemporal scale: broad spatial extents and long temporal windows amplify environmental filtering, whereas fine scales and early successional phases accentuate competitive and other biotic interactions [15,25,27,33,69].

Notably, competition intensity (CI) had little effect on PD and SR, whereas microtopographic factors such as elevation, aspect, the TRI, and roughness exerted strong effects across all life stages. Elevation’s influence on diversity extended throughout the ontogenetic sequence (Figure 8(b1,b2)), likely because the plot is situated at relatively high elevation, amplifying the importance of climate- and soil-related filtering relative to competition during the early and intermediate stages. For saplings and juveniles, aspect was particularly important (Figure 8(c1,c2)), suggesting light-driven filtering, while for adults, locally adapted root and canopy structures may reduce small-scale sensitivity, making them more responsive to broad environmental gradients such as elevation, roughness, or hydrological connectivity. Adulthood thus reflects the cumulative effects of earlier filtering and competition, with the remaining cohorts mostly comprising functionally conservative groups [6,15,22,42].

Collectively, our results suggest a dynamic, stage-dependent balance: communities exhibit initial phylogenetic clustering (filtering), followed by overdispersion (competition), or stochastic switching between weak clustering and overdispersion [14,28]. Temporally, filtering dominates in saplings and juveniles; competition becomes more important in adults [6,33]. Spatially, microtopographic control is strongest at 2.5–5 m grains but is overridden by macro-gradients at a larger scale, with different factors becoming significant at different stages, raising overall stochasticity [29,42,70]. With niche conservatism, filtering is accentuated for traits such as shade or drought tolerance; when functionally divergent taxa assemble, competitive exclusion drives overdispersion [24,42,60]. Our findings align with the existing literature and highlight a hallmark of subtropical forests: the coexistence of tropical- and temperate-like assembly processes and highly heterogeneous spatial patterns due to complex orography [71,72].

### 4.3. Limitations and Future Directions

This study constructed phylogenetic relationships based on floristic surveys rather than direct molecular data and did not incorporate functional traits as a bridge between the phylogenetic structure and demographic parameters of populations—links likely to be crucial for understanding functional trait divergence in relation to microtopography [68,73]. This limitation should be addressed in future studies. Additionally, this research used tree diameter at breast height (DBH) to classify life-history stages and relied on the “space-for-time” substitution approach to infer successional patterns, aiming to reveal the evolutionary rules of community phylogenetic structure across different developmental stages (e.g., transition from environment-filtering-dominated clustering to competition-dominated dispersion), rather than directly quantifying population dynamic processes such as individual growth rates or mortality. One limitation of this study is the absence of direct integration of functional traits as mediating variables in the analysis.

Looking ahead, a key direction for future research is to focus on functional trait analysis as a bridge between phylogenetic relationships and demographic (population-level) processes. Integrating functional traits into long-term studies will allow us to directly link evolutionary history with species’ ecological strategies and performance, thereby deepening our understanding of the mechanisms underlying forest community assembly and dynamics. Long-term monitoring data (≥10 years) are needed in the future to elucidate individual dynamic mechanisms [6] while simultaneously capturing the influencing factors of episodic disturbances (e.g., extreme storms, frost, fires, wildlife activities, or invasive species), which should be incorporated into future models for in-depth analysis [68,74].

Nonetheless, our data support the critical role of microtopographic heterogeneity in sapling recruitment and juvenile survival [16,31]. Microhabitats such as surface depressions, litter accumulations, and fallen logs can serve as refugia for regeneration or as hotspots for pathogens [13]. North-facing slopes often favor shade-tolerant rarities, while south-facing sites select for species with high transpiration and UV tolerance. For conservation and management, these findings argue for slope- and microhabitat-specific interventions: preserving shade environments for rare shade-tolerant species and minimizing disturbance and maintaining ground cover on sun-exposed slopes to reduce water loss [42,65]. In natural forest management or restoration, we recommend prioritizing the identification and protection of critical microtopographic patches to maximize regeneration-phase diversity. Microtopography not only structures resource distribution but also shapes interaction networks—including competition and facilitation (e.g., mycorrhizal mutualisms) [42,75,76]. Future management should integrate these networks at the community or ecosystem level. Long-term monitoring and integration of functional traits and molecular approaches will further elucidate the coupling of microtopography, phylogeny, and interspecific interaction [24,42]. Monitoring tree survival, growth, and reproduction across microhabitats, combined with molecular or isotopic tracking, can ultimately reveal the intricate dynamics among competition, cooperation, and filtering [24,46].

## 5. Conclusions

This study uniquely integrates phylogenetic structure and high-resolution microtopographic analyses across fine spatial grains (2.5 m, 5 m, and 10 m) to investigate community assembly processes in subtropical evergreen broad-leaved forests of the Yaoluoping National Nature Reserve. Our approach systematically examines how microtopographic heterogeneity, together with biotic interactions, influences phylogenetic and compositional patterns across life stages and spatial scales within such forests. The main novel findings are as follows: (1) We demonstrate that both environmental filtering and interspecific competition operate simultaneously but with varying intensities depending on spatial scale and developmental stage—a pattern clarified by integrating phylogenetic and microtopographic data. (2) High-resolution topographic variables (aspect, roughness, TPI, and TRI) are shown to have significant, scale-dependent influences on community structure, and their interaction with biotic competition is strongest at the finest spatial grains. (3) Our results reveal that competition has the greatest effect on diversity among saplings and juveniles at fine scales, supporting negative density dependence predictions, while environmental filtering becomes increasingly dominant in later life stages. (4) We uncover how contrasting microhabitats (e.g., shaded vs. exposed slopes and ridges vs. depressions) drive multiple successional trajectories, leading to high heterogeneity and a dynamic balance between abiotic filtering and biotic interactions.

We also acknowledge that our study did not incorporate long-term data or functional traits of both aboveground and belowground components, which remains a limitation. Future research combining long-term monitoring, key functional traits, and belowground mutualistic networks (such as mycorrhizal associations) is needed to obtain a more comprehensive understanding of forest community assembly and maintenance mechanisms. Looking ahead, a key direction for future research is to focus on functional trait analysis as a bridge between phylogenetic relationships and demographic processes. Integrating functional traits into long-term studies will allow us to directly link evolutionary history with species’ ecological strategies and performance, thereby deepening our understanding of the mechanisms underlying forest community assembly and dynamics.

## Data Availability

Data are contained within the article and Supplementary Materials.

## Supplementary Materials

The following supporting information can be downloaded at https://www.mdpi.com/article/10.3390/plants14142098/s1, Table S1. Table of corresponding abbreviations of plants in the fixed sample plot of Yaoluoping. Table S2. NRI and NTI of subtropical evergreen broad-leaved forest communities across different life-history stages and sampling scales in Yaoluoping, Anhui. Figure S1. Regional map of the fixed monitoring sample plot in the Yaoluoping National Nature Reserve. Figure S2. Schematic diagram of the diameter at breast height (DBH) measurement protocol. Figure S3. Spatial variation phylogenetic and neighborhood predictors at the whole-plot community scale: SR (a), PD (b), NRI (c), and NTI (d). These maps were generated using the Epanechnikov kernel function of the terra package in R (version 4.4.2), and the intensity values range from light blue (low) to light purple (high).
