# Supplementary material for: Phylogenetic Structure Shifts Across Life-History Stages in Response to Microtopography and Competition in Subtropical Forests
Source: Plants (Basel). 2025 Jul 8;14(14):2098. doi: 10.3390/plants14142098 (PMC12299180; doi:10.3390/plants14142098)

Supplementary Files

Table S1. Table of Corresponding Abbreviations of Plants in the Fixed Sample Plot of Yaoluoping.

Species	Abbreviation	Species	Abbreviation
Acanthopanax gracilistylus	Acanthopanax	*Acer amplum* var. *amplum*	Acer2
Acer anhweiense	Acer8	*Acer elegantulum*	Acer4
Acer grosseri var. *grosseri*	Acer5	*Acer henryi*	Acer3
Acer macrophyllum	Acer9	*Acer nikoense*	Acer1
Acer palmatum var. *palmatum*	Acer7	*Acer pictum* subsp. *mono*	Acer6
Actinidia chinensis	Actinidia	*Aralia chinensis*	Aralia
Betula luminifera	Betula	*Bothrocaryum controversum*	Bothrocaryum
Carpinus cordata var. *chinensis*	Carpinus2	*Carpinus turczaninowii*	Carpinus1
Castanea seguinii	Castanea	*Cerasus serrulata*	Cerasus
Cercidiphyllum japonicum	Cercidiphyllum	*Cercis chinensis*	Cercis
Cladrastis wilsonii	Cladrastis	*Clerodendrum trichotomum*	Clerodendrum
Corylopsis sinensis	Corylopsis	*Corylus heterophylla* var. *sutchuenensis*	Corylus
Cyclobalanopsis myrsinaefolia	Cyclobalanopsis	*Cyclocarya paliurus*	Cyclocarya
Dendrobenthamia japonica var. *chinensis*	Dendrobenthami a	*Deutzia glauca*	Deutzia2
Deutzia ningpoensis	Deutzia1	*Elaeagnus umbellata*	Elaeagnus
Euscaphis japonica	Euscaphis	*Evodia fargesii*	Evodia
Fraxinus insularis	Fraxinus	*Hamamelis mollis*	Hamamelis
Hydrangea chinensis	Hydrangea1	*Hydrangea paniculata*	Hydrangea2
Ilex chinensis	Ilex1	*Ilex cornuta*	Ilex4
Ilex macrocarpa	Ilex3	*Ilex macropoda*	Ilex2
Ilex pedunculosa	Ilex5	*Ilex serrata*	Ilex6
Jasminum sambac	Jasminum	*Lespedeza buergeri*	Lespedeza
Lindera erythrocarpa	Lindera6	*Lindera fruticosa* var. *fruticosa*	Lindera5
Lindera glauca	Lindera3	*Lindera obtusiloba*	Lindera1
Lindera praecox	Lindera4	*Lindera reflexa*	Lindera2
Magnolia pilocarpa	Magnolia	*Mallotus japonicus* var. *floccosus*	Mallotus
Malus hupehensis	Malus	*Meliosma flexuosa*	Meliosma2
Meliosma oldhamii	Meliosma1	*Meliosma veitchiorum*	Meliosma3
Morus australis	Morus	*Padus brachypoda*	Padus3
Padus obtusata	Padus2	*Padus racemosa*	Padus1
Philadelphus incanus	Philadelphus	*Photinia beauverdiana*	Photinia2
Photinia beauverdiana var. *brevifolia*	Photinia3	*Photinia parvifolia*	Photinia4
Photinia serrulata	Photinia5	*Photinia villosa*	Photinia1
Phyllanthus glaucus	Phyllanthus	*Picrasma quassioides*	Picrasma

Species	Abbreviation	Species	Abbreviation
Pinus taiwanensis	Pinus	*Platycarya strobilacea*	Platycarya
Pterostyrax corymbosus	Pterostyrax	*Pyrus betulaefolia*	Pyrus
Quercus aliena	Quercus2	*Quercus serrata*	Quercus3
Quercus serrata var. *brevipetiolata*	Quercus1	*Rhamnus globosa*	Rhamnus2
Rhamnus parvifolia	Rhamnus1	*Rhamnus wilsonii*	Rhamnus3
Rhododendron fortunei	Rhododendron2	*Rhododendron mariesii*	Rhododendron 3
Rhododendron polycladum	Rhododendron4	*Rhododendron simsii*	Rhododendron 1
Rhus chinensis	Rhus	*Salix integra*	Salix1
Salix matsudana	Salix2	*Saxifraga stolonifera*	Saxifraga
Sorbus alnifolia	Sorbus1	*Sorbus hupehensis*	Sorbus2
Spiraea cantoniensis	Spiraea2	*Spiraea salicifolia*	Spiraea1
Stachyurus chinensis var. *chinensis*	Stachyurus	*Staphylea bumalda*	Staphylea
Stewartia sinensis	Stewartia	*Styrax japonicus*	Styrax1
Styrax obtassia	Styrax2	*Symplocos paniculata*	Symplocos
Tilia japonica	Tilia1	*Tilia miqueliana*	Tilia2
Tilia tuan	Tilia3	*Torreya grandis* ' Merrillii '	Torreya
Toxicodendron succedaneum	Toxicodendron	*Viburnum betulifolium*	Viburnum2
Viburnum sympodiale	Viburnum1	*Weigela japonica* var. *sinica*	Weigela

Table S2. NRI and NTI of Subtropical Evergreen Broad-Leaved Forest Communities Across Different Life-History Stages and Sampling Scales in Yaoluoping, Anhui.

Tree Life Stages	Sampling Scale	NRI	NTI
All	2.5m×2.5m	-0.18500	-0.17000
	5m×5m	-0.27760	-0.17100
	10m×10m	-0.28335	-0.16359
Sapling	2.5m×2.5m	0.1379 ***	0.1047 ***
	5m×5m	0.1538 ***	0.0803 ***
	10m×10m	0.18025 ***	0.02104
Juvenile	2.5m×2.5m	-0.328	-0.208
	5m×5m	-0.2288	-0.1544
	10m×10m	-0.27648	-0.05430
Adult	2.5m×2.5m	-0.7525	-0.7335
	5m×5m	-0.6594	-0.5969
	10m×10m	-0.7986	-0.5764

*** : $P < 0.001$

Figure S1. Schematic diagram of the layout of sampling units in the plot, with four-digit codes representing stake numbers.

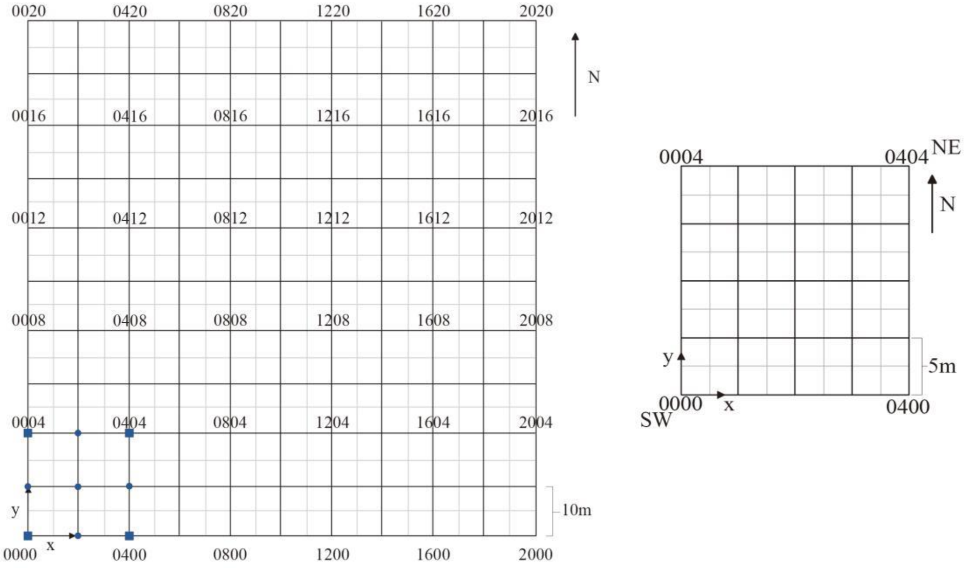

Figure S2. Schematic diagram of the diameter at breast height (DBH) measurement protocol

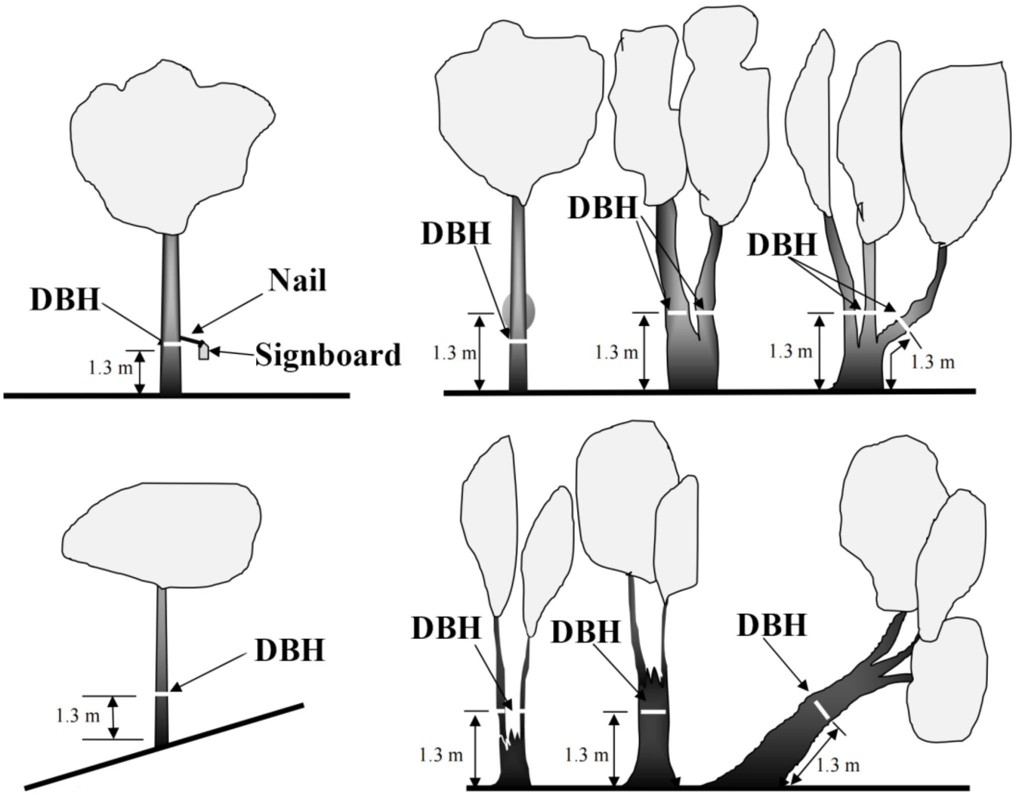

Figure S3. Spatial variation of species richness (SR, **a**) and phylogenetic index: PD (**b**), NRI (**c**),NTI (**d**). These maps were generated using the Epanechnikov kernel function of the terra package in R, and the intensity values range from light blue (low) to light purple (high).

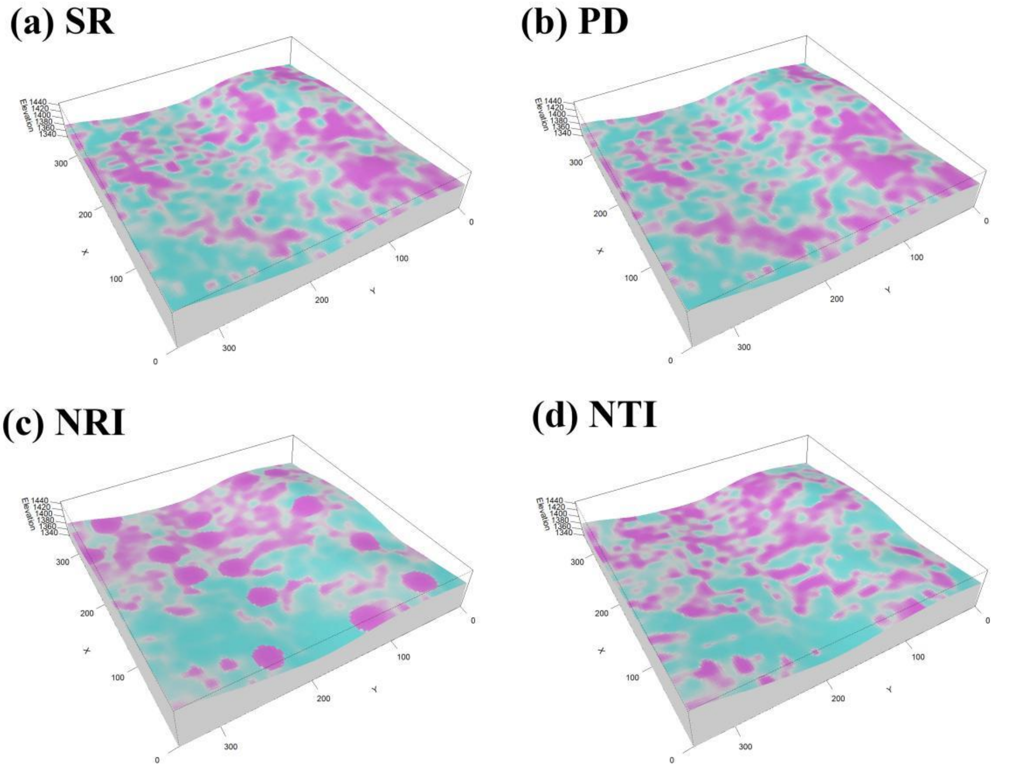

Supplement: Supplementary file 1 [file plants-14-02098-s001.zip › plants-3678608-supplementary.pdf]
